# Supplementary material for: Chromothripsis during telomere crisis is independent of NHEJ, and consistent with a replicative origin
Source: Genome Res. 2019 May;29(5):737–49. doi: 10.1101/gr.240705.118 (PMC6499312; doi:10.1101/gr.240705.118)
Supplement: Supplemental Material [file supp_gr.240705.118_Supplemental_file_1.zip › contigs/annotated_contigs/DB39/contig.2.DB39_length_628_mean_cov_3.82165605096.docx]

**DB39_length_628_mean_cov_3.82165605096**

```
TGAAATTAATAAAGGGAAAGAATTTATGAGGCTATGATTTTGTATTTTGTATCTGAACTATATATACTGTTTTTCTGTCAGTCTTTGTG
 >chr13:85773844-85774215 - E=1e-211
TTTTATGAAGATATTCTGGGTAGTCATCACATGGACATGCTGATTAGGATGCATTCATTGACACCTCTGTTTTTGGACTCTATAAAGGT

CAGCCAAACATATGGCCCCAAACAGCTTATATTCCTAAATCTTGCCAAGTTTGGAAAATTGAACCTACATTGTATATCAGAAAAAGACA

ATTTTATTCAGCAACTCATAAGTATTTTGTACAGACCAGGGTACTGTACAATATTATCTTCTAACTTAATAGCCCAGTTTTCTCTGTGG

AAGCCAGGTATGCT|T|GATCCATGGACTGTCAGCAGGAATATCAGCTGTTAGTGTGAGAGAAAGGCAAAATTTCAAGTCCCACCTCAC
               >chr13:85775384-85775642 - E=2e-143
GTTTTTAAATCAGAATCTAAATGTTAACAAGATGGCCAGTGGTACCTATGTGCATTAAATTTTGAGCAGAGCTGGAATTTGCAATAAGT

TAAACTGTTGCTCTCACAAGCATCATTTTTTAAACTCTTGATTTTCAATTCTTCATTTTGTGGTGACAATGACAAAAAAGACTTGCAGT

TTAACTG
```
